# Post Neck Dissection Chyle Leak Repair with Omohyoid Flap - A Novel Operative Technique

**DOI:** 10.1007/s12070-025-05468-7

**Published:** 2025-04-28

**Authors:** Vidula Mestry, Prathamesh S. Pai

**Affiliations:** 1 Department of Surgical Oncology, Division of Head and Neck Surgery, Tata Memorial Centre, ACTREC, Mumbai, India; 2 Punyashlok Ahilyadevi Holkar Head and Neck Cancer Institute of India (HNCII), Mumbai, India

**Keywords:** Chyle leak, Neck dissection, Omohyoid flap

## Abstract

Chyle leak is a potentially serious complication that can occur after neck dissection. It is characterized by the leakage of milky fluid in the neck area which occurs due to thoracic or lymphatic duct injury. Chyle leak can lead to severe metabolic and wound related complications. Intraoperative identification and prompt repair can prevent further complications. Traditional surgical repair method is ligating or oversewing the duct with surrounding soft tissue, but in cases where extensive level IV neck dissection is performed, alternative techniques are required. This article describes a novel technique of using omohyoid muscle flap for surgical repair of chyle or lymphatic leak after neck dissection. Underlying principle of the method involves employing a vascularized flap of the omohyoid muscle to exert pressure on the duct to compress it against the prevertebral muscles, while also sealing the location of the chyle leak. The muscle is affixed in position by suturing it to the carotid fascia and the fascia of the prevertebral region. This approach expedites the healing process and offers efficient control. This technique can be utilized when conventional approaches prove ineffective or during surgical exploration for continual chyle leaks. Proposed technique facilitates prompt intraoperative control of chyle or lymphatic leakage in the level IV region following neck dissection. Inferiorly based omohyoid flap can be effectively used with the described technique for post neck dissection chyle leak repair without giving additional morbidity and with lesser learning curve for surgeons.

## Introduction

Chyle leak, also known as chylous fistula, is a relatively rare but serious complication that can occur after neck dissection [1]. Chyle is a milky bodily fluid containing emulsified fats (Chylomicrons) and lymph that is carried from intestinal lacteals to venous circulation in neck via thoracic duct [2–4]. Chyle leak is characterised by intraoperative extravasation of milky or clear fluid in chyle duct area of neck or milky drain fluid or milky discharge through neck incision in postoperative period [5].

Incidence of chyle leak after neck dissection is 2–8% [6]. Chyle leak is synonymous with thoracic duct injury on left side, however Right sided chyle leak may be seen in 25% cases [2, 6]. Occasional splitting of thoracic duct into right and left branches in its upper part, with left branch terminating as usual course and right branch terminating in right sided venous system or right lymphatic duct explains right sided chyle leak [2].

Consequences of untreated chyle leak can be severe. Chyle in wound changes local biochemical milieu and incites an intense pro-inflammatory reaction, leading to wound complications and flap compromise in case of microvascular reconstruction [7]. Chyle leak causes hypoproteinaemia, hypovolemia, dyselectrolytemia and dehydration [2, 8] which complicate further recovery. Chylothorax is a potential complication in high output leaks and injury to thoracic duct low down in neck.

Intraoperative detection and control of chyle leak by a proper repair technique prevents further complications [2, 9, 10]. Traditionally chyle leak is surgically managed by ligating duct or oversewing the duct or ductules with surrounding soft tissue. Whenever extensive level IV neck dissection is performed, local level IV connective tissue may not be available for ligating or oversewing the duct. Use of vascularised muscle flap is effective treatment of chyle leak repair [11]. Omohyoid muscle is locally available indispensable muscle which can be used as vascularised flap [12]. We describe novel technique of using omohyoid flap for surgical repair of chyle or lymphatic leak after neck dissection.

## Materials and Methods

### Technique of Chyle Leak Repair with Omohyoid Flap

Post neck dissection, chyle or lymphatic leak is confirmed with a positive pressure ventilation against closed expiration valve or with Cernea’s manoeuvre [13]. Described technique is performed if leak is persistent after conventional ligation or clipping. Carotid sheath over inferolateral aspect of IJV is gently dissected and cut to provide anchor for the muscle. Superior belly of omohyoid is divided from hyoid attachment and sutured to dissected carotid sheath medially and prevertebral fascia laterally.

**Fig. 1:**
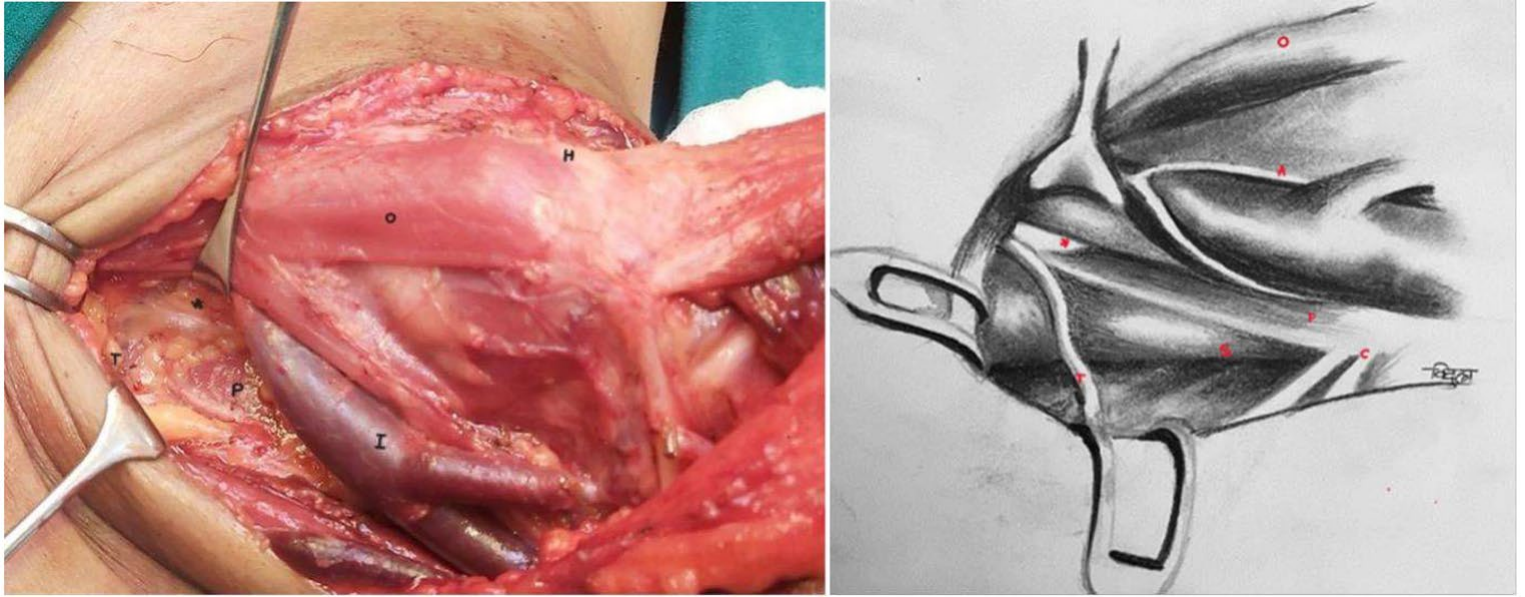
Thoracic duct in inferomedial part of level IV. (Annotations *- Thoracic duct, I- IJV, H-Hyoid bone, O-Omohyoid muscle, T-Transverse cervical artery, C- Cervical Plexus, P- Phrenic nerve, A- Ansa Cervicalis, S- Scalene anterior muscle)

**Fig. 2:**
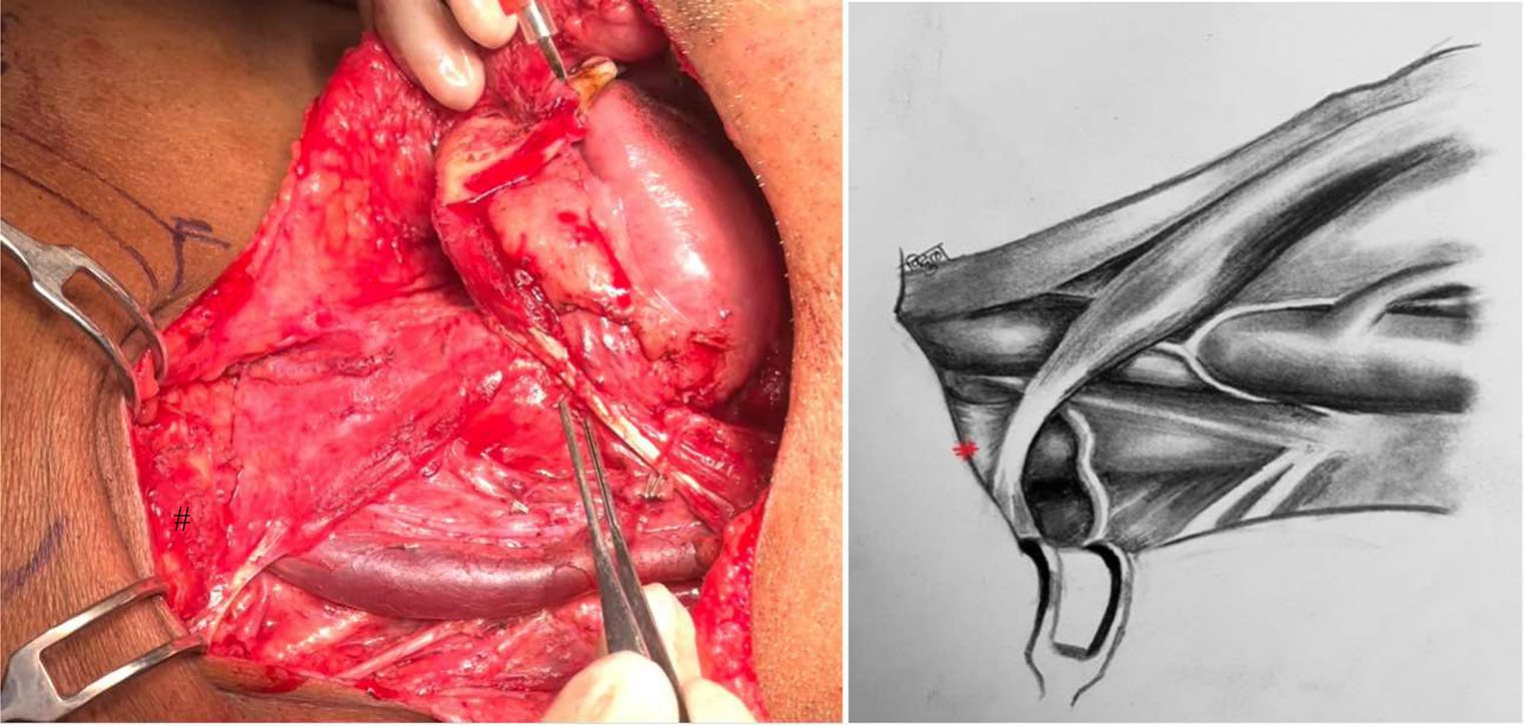
Omohyoid muscle exposed as medial boundary of neck dissection. (Annotation # Clavicular attachment of omohyoid)

**Fig. 3:**
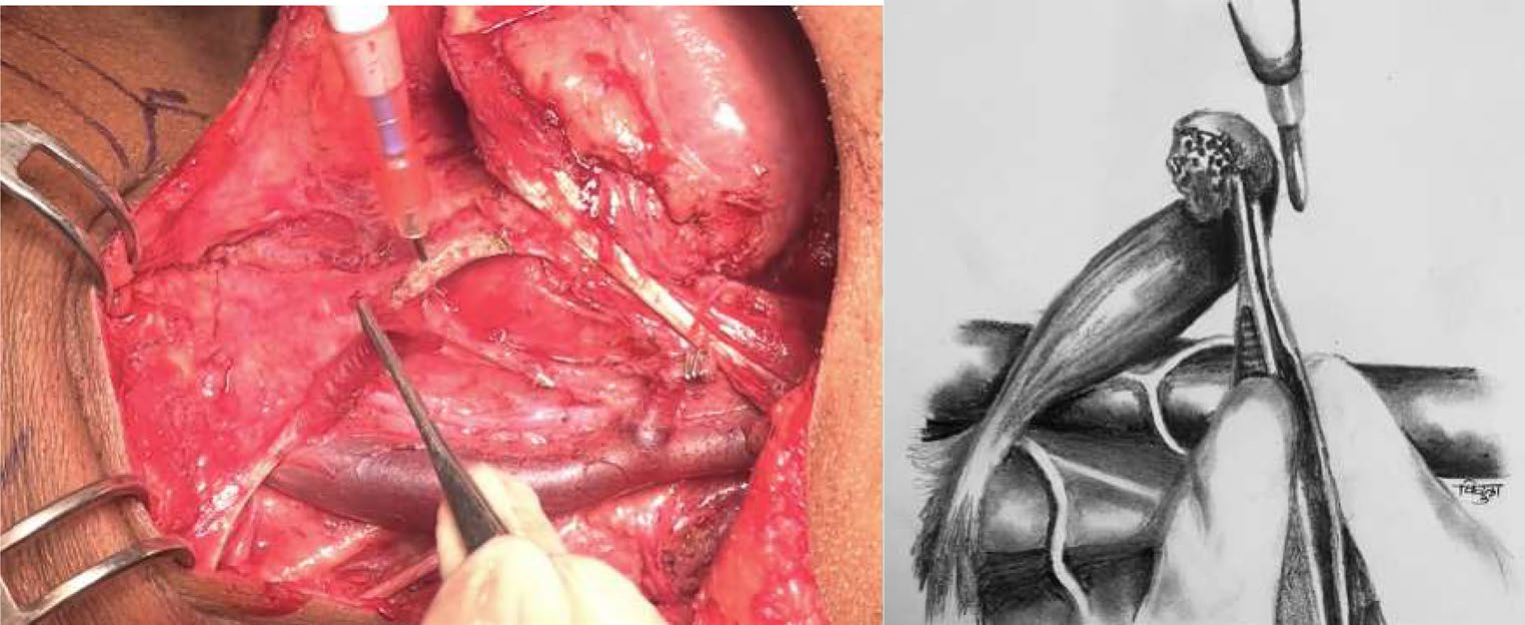
Omohyoid muscle is transacted at its attachment to hyoid. Superior belly is mobilised by dissecting off its bed upto intermediate tendon, where its attached to clavicle

**Fig. 4:**
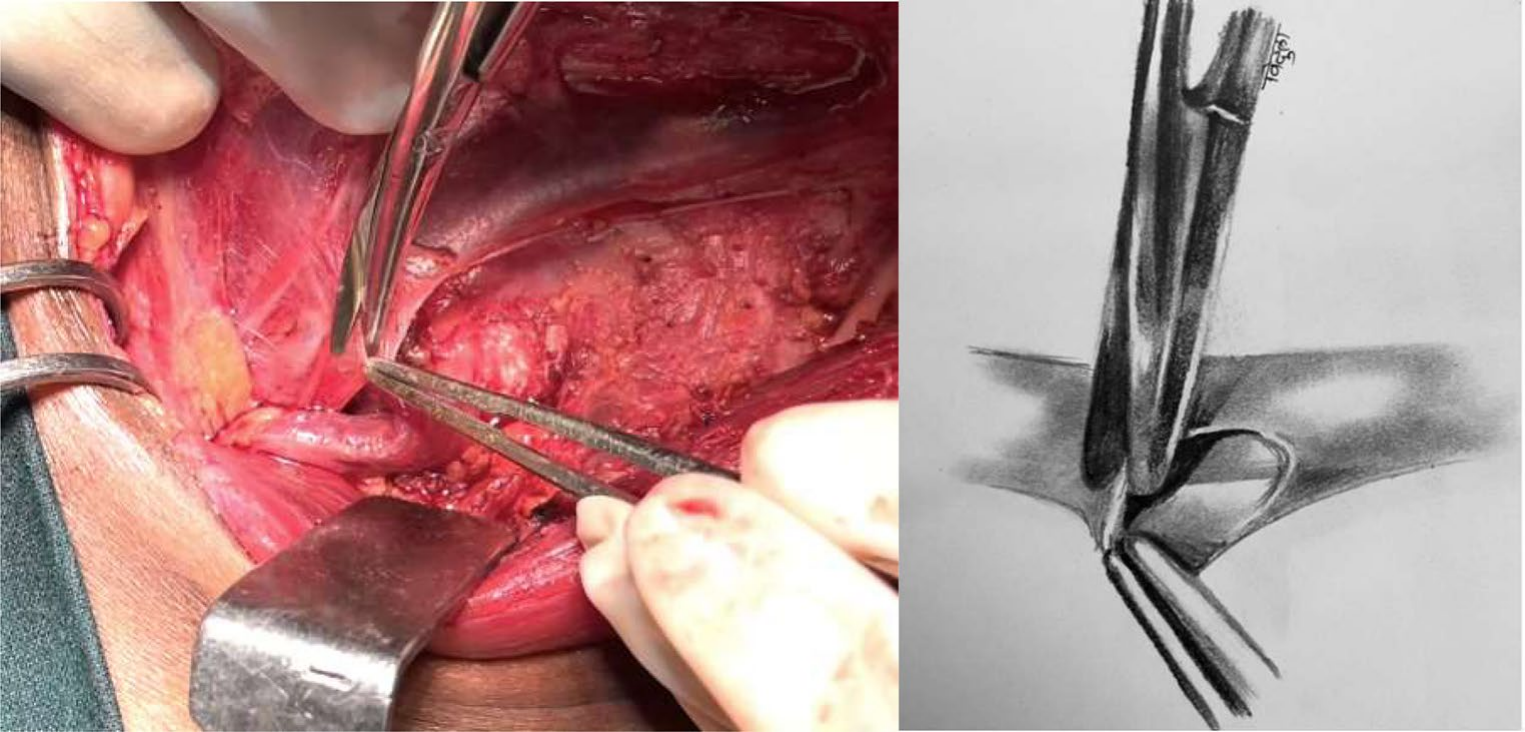
Fascia over inferolateral aspect of lower 1/3rd of IJV is gently dissected off venous adventitia to get a thin fascial layer for anchoring omohyoid muscle

**Fig. 5:**
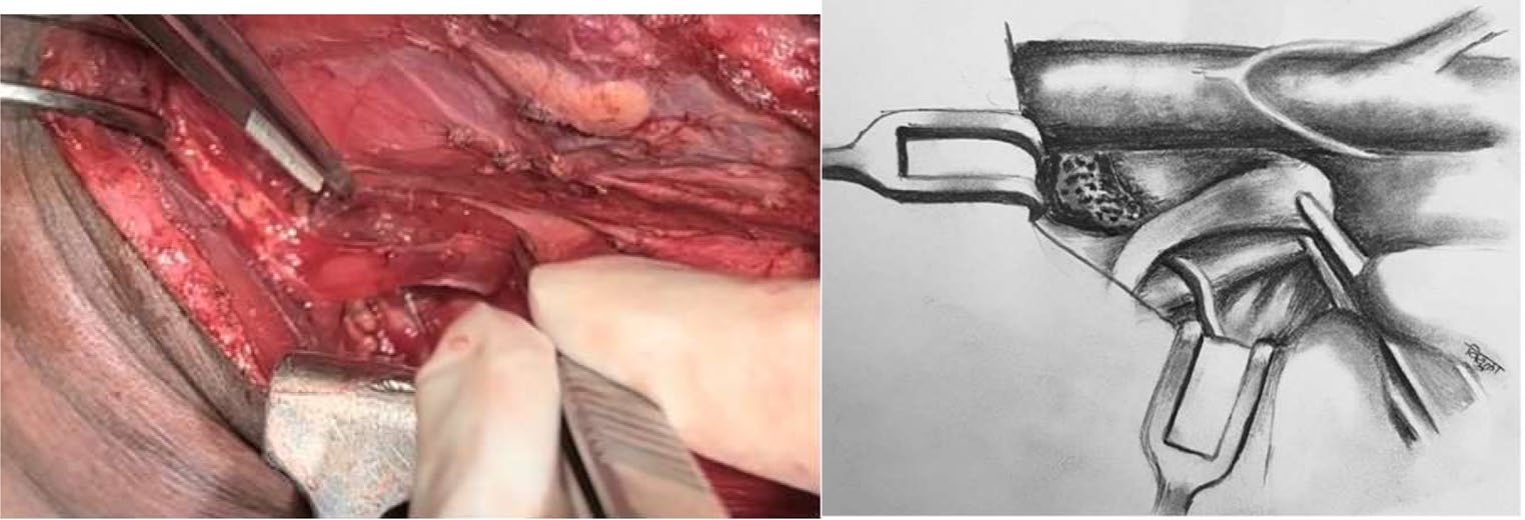
Omohyoid muscle is folded in such a way that superior cut end fills chyle / lymphatic duct area

**Fig. 6:**
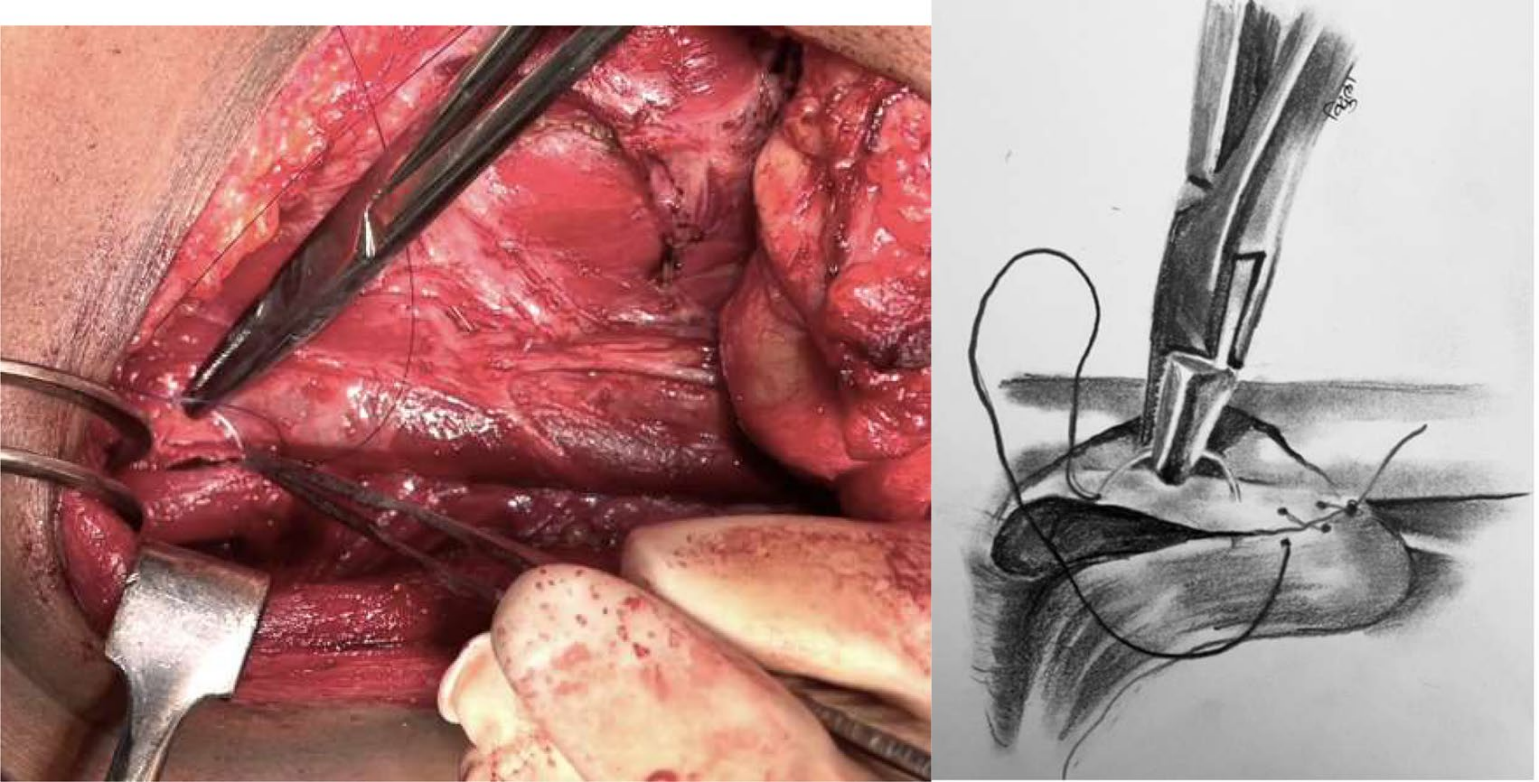
Dissected fascia over inferomedial aspect of IJV is sutured to medial border of omohyoid muscle with 4 − 0 PDS suture (round body needle) in continuous manner upto clavicular attachment of omohyoid. To note, raw cut end is obliterating inferomedial chyle leak area

**Fig. 7:**
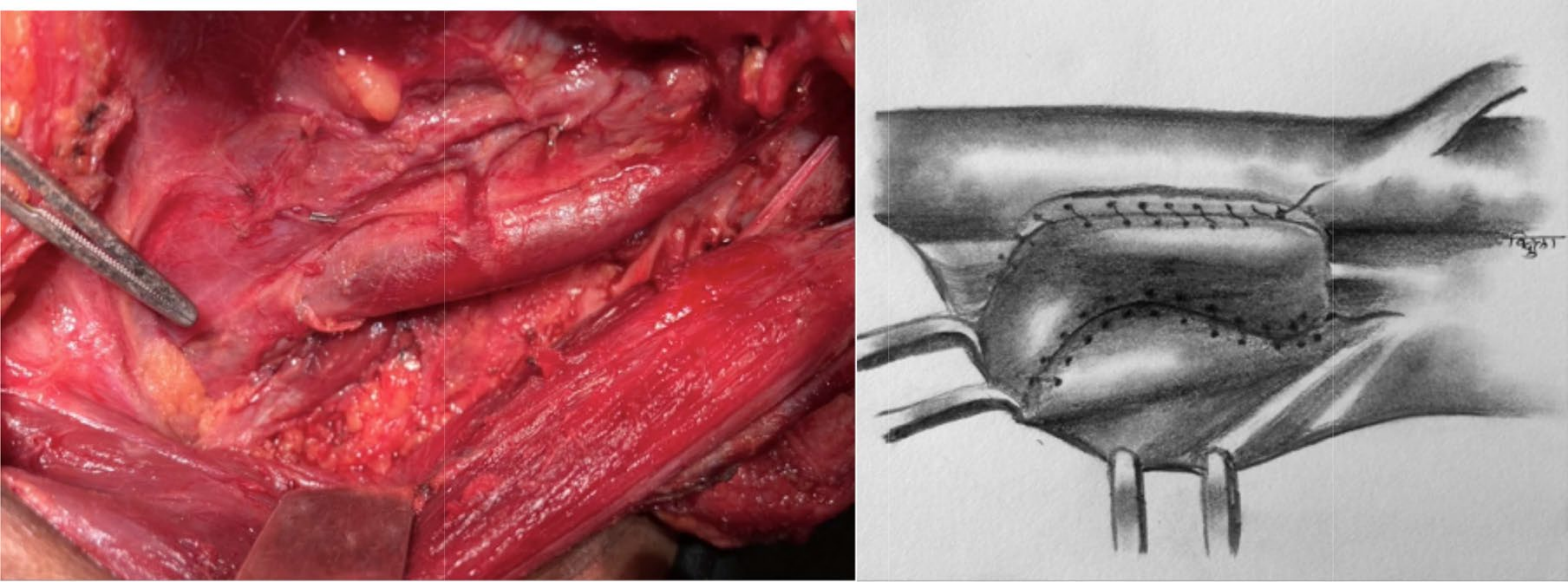
Lateral border of omohyoid is sutured to prevertebral fascia over scalene anterior muscle or loose fascia over lying brachial plexus lateral to lateral border of scalene anterior muscle. Care should be taken to identify phrenic nerve and not to include it in suturing

This is pictorial description of novel and systematic surgical technique of inferiorly based omohyoid flap for post neck dissection chyle leak repair, in simple and reproducible steps. All pictures and illustrations are in left side of neck with patient in supine position and head end on right side (Figs. 1, 2, 3, 4, 5, 6 and 7).

## Discussion

Thoracic duct enters left root of neck in prevertebral area between longus colli and scalene anterior muscles. It lies lateral to oesophagus, posterior to common carotid artery, anterior to thyrocervical trunk and phrenic nerve, ascends 3–4 cm cranial to clavicle, turns anterolaterally to open into venous system at the confluence of internal jugular vein and subclavian vein (Pirogoff’s angle) [3, 4]. Anatomy of thoracic duct at root of neck is relatively constant. Thoracic duct can be ligated, compressed against prevertebral muscles and plugged with vascularised muscle in this area to achieve effective control of chyle leak.

Principle of the described technique is to use vascularised omohyoid muscle flap to compress duct against prevertebral muscles and to plug chyle leak area with vascularised tissue to hasten healing. Muscle can be secured in position, medially by suturing to carotid sheath which can be easily dissected off from venous adventitia of IJV and laterally to prevertebral fascia. This technique can be used if chyle leak is persistent after conventional ligation or in surgical exploration for chyle leak after neck dissection.

Carotid sheath is fibrous condensation of deep cervical fascia that extends from skull base to mediastinum and acts as conduit for common and internal carotid artery, internal jugular vein (IJV) and lower cranial nerves [14, 15]. As noted by Barlow in 1936, carotid sheath over IJV is loosely adherent to venous adventitia [14, 15] thus can be easily dissected off the vein.

Omohyoid is an infrahyoid strap muscle which has superior and inferior belly, connected by intermediate tendon which has fascial attachment to clavicle [3]. Inferior belly originates from upper border of scapula, near scapular notch (occasionally from superior transverse scapular ligament) and inserts onto intermediate tendon [3]. Superior belly originates from intermediate tendon and inserts on body of hyoid bone [3]. Superior belly receives blood supply from superior and inferior thyroid arteries and inferior belly receives blood supply from transverse cervical artery with considerable anastomosis within the muscle [16, 17]. Pertaining to segmental blood supply with intramuscular anastomosis, muscle retains vascularity even if one end of muscle is detached. Omohyoid muscle overlies area of thoracic duct thus unlike other local muscle flaps, less mobilisation is needed and there is least chance of muscle retracting back to its normal position.

In case of repair for high output leaks, x ray chest should be done after commencing oral feeds to check for any collection in the pleural space. Fat free diet is usually not required post repair. Omohyoid muscle flap, when used with above mentioned technique, provides immediate intraoperative control of chyle leak in level IV area and effective in controlling chyle leak during postoperative period.

## Conclusion

Using omohyoid muscle flap has several advantages.

- It is locally available muscle which is exposed after neck dissection.
- It has segmental blood supply- superior, inferior thyroid arteries and transverse cervical artery [3, 16, 17], with intramuscular anastomoses., thus remains vascularised when one end is detached.
- Function of muscle is to depress hyoid post elevation [3], which is relatively indispensable.
- No donor site morbidity unlike with use of other muscle flaps like pectoralis minor myocutaneous flap.
- It overlies thoracic/lymphatic duct area thus extensive mobilisation is not required and tends to remain in place without chance of retraction to site of original attachment.

Inferiorly based omohyoid flap can be effectively used with described technique for post neck dissection chyle leak repair without giving additional morbidity and with lesser learning curve.
